# 肺大细胞神经内分泌癌22例临床分析

**DOI:** 10.3779/j.issn.1009-3419.2016.02.05

**Published:** 2016-02-20

**Authors:** 哲 钱, 瑛 胡, 华 郑, 宇杰 董, 群慧 王, 宝兰 李

**Affiliations:** 1 101149 北京，首都医科大学附属北京市胸科医院，北京市结核病胸部肿瘤研究所综合科 Department of General Medicine, Beijing Chest Hospital, Capital Medical University/Beijing Tuberculosis and Thoracic Tumor Research Institute, Beijing 101149, China; 2 101149 北京，首都医科大学附属北京市胸科医院，北京市结核病胸部肿瘤研究所病理科 Department of Pathology, Beijing Chest Hospital, Capital Medical University/Beijing Tuberculosis and Thoracic Tumor Research Institute, Beijing 101149, China

**Keywords:** 肺肿瘤, 临床特征, 免疫组化, 预后, Lung neoplasms, Clinical features, Immunohistochemical, Prognosis

## Abstract

**背景与目的:**

肺大细胞神经内分泌癌是肺原发恶性肿瘤的一种少见类型，由于其特殊的生物学行为、复杂的病理学标志、多样的影像学表现及欠佳的治疗疗效，亟待进行临床探讨。本研究旨在分析肺大细胞神经内分泌癌的临床资料，为进一步提高其诊治水平提供依据。

**方法:**

回顾性分析22例肺大细胞神经内分泌癌患者的临床特征、诊治情况及预后。

**结果:**

肺大细胞神经内分泌癌好发于有大量吸烟史的老年男性，临床表现以咳嗽、咳痰、咯血、胸痛为主。计算机断层扫描（computed tomography, CT）表现以周围型肿块为主，可伴不均匀强化和坏死。免疫组化神经内分泌分化标志物Syn、CgA和CD56的阳性表达率分别为72.7%、68.2%和68.2%。17例行手术治疗，术后10例接受辅助治疗，5例行姑息化疗。单因素分析提示吸烟指数（*P*=0.029）、淋巴结转移（*P*=0.034）、肿瘤-淋巴结-转移（tumor-node-metastasis, TNM）分期（*P*=0.005）、治疗方法（*P*=0.047）、术后辅助化疗（*P*=0.014）是预后的影响因素，多因素分析提示淋巴结转移（*P*=0.045）及术后辅助化疗（*P*=0.024）是预后的影响因素。

**结论:**

肺大细胞神经内分泌癌缺乏特异性的临床表现，确诊依赖术后病理，各种治疗疗效欠佳。淋巴结转移状态及术后辅助化疗是影响预后的重要因素。

## Abstract (en)

**Background and objective:**

Pulmonary large cell neuroendocrine carcinoma (LCNEC) is a rare primary malignant tumor. Due to poor understanding of its biologic behaviors, pathological features, image manifestations and clinical effects, clinical study is urgent. Analysis of clinical data of pulmonary LCNEC, in order to improve the clinical diagnosis and treatment.

**Methods:**

Retrospective analysis of 22 pulmonary LCNEC cases of clinical features, diagnosis, treatments and prognosis.

**Results:**

Pulmonary large cell neuroendocrine carcinoma occurs in older men with heavy smoking history., clinical symptoms are cough, sputum, hemoptysis, and chest pain. Computed tomography (CT) features are peripheral mass mainly, accompanied by heterogeneous density and necrosis. Immunohistochemical neuroendocrine differentiation markers Syn, CgA and CD56 positive expression rates were: 72.7%, 68.2% and 68.2%, respectively. 17 patients underwent surgical treatment, 10 patients received adjuvant therapy, 5 underwent palliative chemotherapy. Univariate analysis indicated that smoking index (*P*=0.029), lymph node metastasis (*P*=0.034), tumor-node-metastasis (TNM) stage (*P*=0.005), treatment (*P*=0.047), postoperative chemotherapy (*P*=0.014) are prognostic factors. Multivariate analysis showed that lymph node metastasis (*P*=0.045) and postoperative chemotherapy (*P*=0.024) are prognostic factors.

**Conclusion:**

Pulmonary LCNEC is lack of specific clinical symptoms, and its pathological diagnosis depends on postoperative specimens, poor efficacy of various treatments is its current situation. Lymph node metastasis and postoperative chemotherapy are important prognostic factors.

肺大细胞神经内分泌癌（large cell neuroendocrine cancer, LCNEC）是由Travis等^[1]^于1991年首次命名的肺恶性肿瘤，是一种临床少见的肺原发恶性肿瘤，其发病率仅占肺癌病例的2.1%-3.5%^[2]^。此前的世界卫生组织（World Health Organization, WHO）肺癌病理分类将其归于大细胞癌，但最新版WHO（2015）肺癌病理分类已将LCNEC调整出大细胞癌这一分类，而与小细胞肺癌（small cell lung cancer, SCLC）及类癌（typical carcinoid, TC）等共同归于神经内分泌肿瘤，这一病理分类上的调整可能会给临床诊疗工作带来新的指导^[3]^。恶性程度高、误诊率高、术前诊断困难及治疗效果差是肺LCNEC重要的临床特点，而发病率低、缺乏大样本数据支持的诊疗规范更让肿瘤科医生因认知不足而不能有效地对肺LCNEC患者进行诊疗。国内外对肺LCNEC的研究相对较少，为了更深入地认识肺LCNEC，我们设计了本研究，旨在回顾性分析我院的肺LCNEC患者的临床特点及诊疗方法，为进一步对肺LCNEC临床诊疗探索提供依据。

## 1 资料与方法

### 1.1 一般资料

收集2005年1月-2015年6月于我院就诊经病理确诊的肺LCNEC患者22例，男性19例，女性3例，年龄49岁-78岁，平均年龄62.82岁。吸烟患者21例，吸烟指数0-3, 000，平均吸烟指数931.82。分期标准采用国际肺癌研究协会2009年第7版肿瘤-淋巴结-转移（tumor-node-metastasis, TNM）分期系统：Ⅰa期4例，Ⅰb期3例，Ⅱa期3例，Ⅱb期1例，Ⅲa期3例，Ⅲb期5例，Ⅳ期3例。

### 1.2 诊断方法

22例患者均行胸部增强计算机断层扫描（computed tomography, CT）检查发现肺内占位性病变。有7例患者气管镜下见新生物予以活检，有1例确诊为肺LCNEC，其余有1例考虑非小细胞肺癌（non-small cell lung cancer, NSCLC），2例考虑SCLC，1例考虑大细胞肺癌，1例考虑为重度不典型增生，另有1例考虑为肺组织慢性炎症。有2例患者行肺穿刺活检检查，其中1例为轻度异形，1例为倾向大细胞肺癌。17例患者经手术大病理确诊为肺LCNEC，另有4例患者经浅表淋巴结活检确诊。病理诊断参考2004年WHO提出的病理诊断标准，包括：①神经内分泌形态；②高细胞分裂比例；③坏死；④NSCLC细胞学特征；⑤嗜铬素A（chromogranin A, CgA）、突触素（synaptophsin, Syn）和CD56/神经细胞粘附分子三种免疫组化标记至少一种阳性或电镜下可见神经内分泌颗粒。

### 1.3 治疗方法

手术切除17例患者，其中2例行全肺切除术，均为左全肺切除。11例行肺叶袖式切除，4例行肺叶楔形切除，无单纯探查手术。接受手术治疗的患者无围手术期死亡。2例患者行术前新辅助化疗，采用紫杉醇联合铂类化疗方案。10例患者接受术后辅助化疗，分别应用培美曲塞、多西他赛、吉西他滨、紫杉醇等联合铂类等化疗方案，其中行4例接受6周期术后辅助化疗、5例接受4周期术后辅助化疗、1例接受2周期术后辅助化疗，4例联合局部姑息性放疗（1例全脑、1例腰椎、2例纵隔淋巴结）。5例不可手术的晚期患者，分别接受培美曲塞、吉西他滨、紫杉醇、依托泊苷等联合铂类方案化疗。

### 1.4 疗效评估标准

按照实体瘤疗效评价标准（Response Evaluation Criteria in Solid Tumors, RECIST）1.1版本进行评估。

### 1.5 随访

对全部22例患者临床资料进行回顾性研究，通过查阅病历资料及电话随访，随访内容包括：性别、年龄、首发症状、吸烟指数、影像学表现、确诊方式、TNM分期、治疗方式及生存时间。随访期为自确诊之日至末次随访日或死亡时间，生存时间从确诊之日算起，随访截止时间为2015年10月31日。

### 1.6 统计学方法

采用SPSS 19.0统计软件分析数据，*Kaplan*-*Meier*法计算生存率，组间差异采用*Log*-*rank*检验；*Cox*比例风险回归模型对患者性别、年龄、吸烟指数、淋巴结转移、TNM分期及治疗方法等进行单因素或多因素预后分析。以*P* < 0.05为差异有统计学意义。

## 2 结果

### 2.1 临床表现

全部22例患者中，首发症状为咳嗽、咳痰者8例，咯血2例，胸痛2例，气胸2例，发热1例。有6例患者无明显症状，为常规体检发现肺内占位就诊。另有1例患者表现为关节肿痛，其他伴随症状有体重减轻、头晕等。发现首发症状到首次住院病程为1周-6个月不等。吸烟患者有21例，吸烟指数均大于400，最高达3, 000，平均931.8。无一例患者合并其他肿瘤（表 1）。

**1:** 22例肺LCNEC患者的临床特征及单因素生存分析结果 Basic clinical features and single factor analysis of prognosis of 22 cases with pulmonary LCNEC

Clinical features	Cases	Percentage（%）	*P*
Gender			0.257
Male	19	86.4	
Female	3	13.6	
Age (year)			0.911
< 60	8	36.4	
≥60	14	63.6	
Smoke index (stick-year)			0.029
0-800	13	59.1	
801-3, 000	9	40.9	
CT location			0.098
Central type	6	27.3	
Peripheral type	16	72.7	
N stage			0.034
N0	9	40.9	
N1	2	9.1	
N2	7	31.8	
N3	4	18.2	
TNM stage			0.005
Ⅰ	7	31.8	
Ⅱ	4	50.0	
Ⅲ	8	36.4	
Ⅳ	3	13.6	
Treatment method			0.047
Operation	17	77.3	
Palliative chemotherapy	5	22.7	
Postoperative chemotherapy			0.014
Yes	10	58.8	
No	7	41.2	
LCNEC: large cell neuroendocrine cancer; CT: computed tomography; TNM: tumor-node-metastasis.			

### 2.2 胸部影像学及气管镜镜下表现

接受治疗前所有患者均行胸部增强CT检查，肿块位于左肺10例，右肺12例。周围型16例，中心型6例，肿瘤最大径1.5 cm-8.3 cm，平均3.7 cm。CT上主要表现为伴分叶状及毛刺的肿块影，增强后均可见不均匀强化，部分伴有坏死，有2例呈空洞型病变。其中11例伴有肺门或纵隔淋巴结增大，3例可见阻塞性肺不张，1例可见支气管缩窄，2例可见胸膜牵拉，1例可见胸腔积液。

在接受了气管镜检查的17例患者中，镜下可见气管内新生物7例，可见粘膜病变的有3例。全组患者原发肿瘤多位于外周（16/22），因此有7例（41.1%）气管镜下无明显异常。

### 2.3 组织病理学及免疫组化特点

本研究中22例患者均行病理与免疫组化检查（图 1）。其中17例手术大体标本，肉眼观切面可呈灰白色或灰褐色，边界欠清，质地较硬，可伴有出血或坏死。全组22例组织病理光镜下表现：多呈巣状、片块状分布，可显示栅栏状、菊团样或梁状结构。肿瘤细胞体积大，胞浆丰富，核大深染，核仁明显，染色质呈细颗粒状或空泡状，多伴核分裂象，大部分可见大片状坏死。免疫组化提示：Syn表达阳性率54.5%，弱阳性率18.2%；CgA表达阳性率36.4%，弱阳性率31.8%；CD56表达阳性率40.9%，弱阳性率27.3%（表 2）。

**1:**
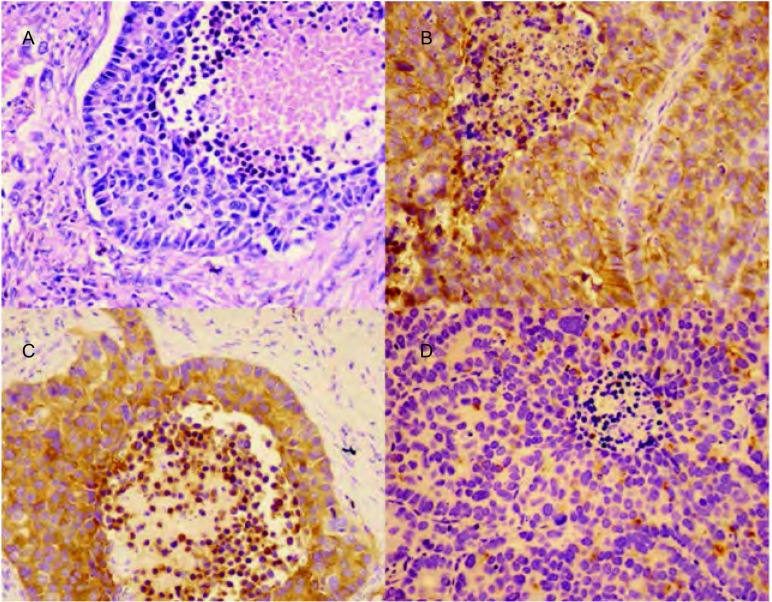
病理与免疫组化检查。A：肿瘤组织内可见巨大肿瘤细胞（HE染色，×200）；B：免疫组化标记Syn呈阳性表达（×200）；C：免疫组化标记CgA呈阳性表达（×200）；D：免疫组化标记CD56呈阳性表达（×200）。 Pathology and immunohistochemistry. A: Tumor cells could be seen in tumor tissue (HE staining, ×200); B: Immunohistochemical staining with Syn (×400); C: Immunohistochemical staining with CgA (×200); D: Immunohistochemical staining with CD56 (×200). CgA: chromogranin A; Syn: synaptophsin.

**2:** 22例LCNEC免疫组化结果 Immunohistochemistry results of 22 cases with pulmonary LCNEC

Results	Syn	CgA	CD56	TTF-1
-	6	7	7	12
+	4	7	6	2
++	12	8	9	8
Positive	16	15	15	10
TTF-1: thyroid transcription factor-1.				

### 2.4 疗效及预后

截止2015年10月31日，22例患者随访率100%，共随访1.0个月-77.3个月，中位随访时间21.3个月。至随访截止日期共死亡13例，生存时间2.5个月-77.3个月，中位生存期为16.8个月。1年生存率40.9%，2年生存率18.2%，3年生存率9.1%。单因素分析提示吸烟指数（*P*=0.029）、淋巴结转移（*P*=0.034）、TNM分期（*P*=0.005）、治疗方法（*P*=0.047）、术后辅助化疗（*P*=0.014）是预后的影响因素（表 1），多因素分析提示淋巴结转移（*P*=0.045）及术后辅助化疗（*P*=0.024）是预后的影响因素（表 3）。

**3:** 22例肺LCNEC患者预后的多因素分析 Multivariate analysis of prognostic factors of 22 cases with pulmonary LCNEC

Factors	*P*	RR	95%CI	
			Lower	Upper
Smoking index	0.193	1.418	0.534	5.064
N stage	0.045	3.059	1.239	21.917
TNM stage	0.081	3.998	0.368	25.398
Treatment method	0.611	0.924	0.283	5.772
Postoperative chemotherapy	0.024	0.676	0.256	0.953

## 3 讨论

LCNEC是肺癌的一种少见病理类型，临床上以男性多见，平均发病年龄为60岁，多数患者有大量吸烟史。本研究中男性占86.4%，女性占13.6%，患病年龄49岁-78岁，吸烟者占95.8%，与国内外其他报道的数据接近^[4-6]^。相比较其他类型的肺癌，LCNEC患者的临床表现缺乏特异性，咳嗽、咳痰、咯血、胸痛为常见的表现，首发症状为神经内分泌异常的病例数要明显少于SCLC，本组中有1例患者因双膝、双足趾关节肿就诊，考虑为内分泌异常引起的关节表现。由于起病隐匿，患者就诊时多数已为中晚期。近年来，随着人们常规体检意识的提高，不少患者因体检胸片发现肺内占位就诊，本组中无任何临床表现体检发现的患者有6例，占27.2%。因此，定期体检，尤其是对伴大量吸烟史的老年男性，是肺LCNEC二级预防的最重要手段。

从胸部CT特征上来分析，肺LCNEC更接近于NSCLC：各肺叶均可发生，上叶略多，多为周围型，中央型占比约20%。肿块或结节边界较清，可伴有分叶、毛刺，其内密度欠均匀，可有坏死，但空洞及钙化均不多见，增强扫描后可出现不均匀强化^[7-9]^。本组患者中周围型病变占72.7%，中央型病变占27.3%，形态学特征与文献报道基本一致，多以肿块及结节为主，伴空洞及点状钙化低于10%，其中50%的患者就诊时合并了肺门或纵隔淋巴结肿大，13.7%的患者合并了胸膜病变。

气管镜对诊断肺LCNEC的帮助有限，本组中54.5%的患者气管镜下未见任何改变，其中最主要的原因是肺内病变多为周围型。同时，我们发现经气管镜或肺穿刺活检取材的小标本确诊率很低，本组中仅1例经气管镜活检确诊为肺LCNEC。此外，2例术前行肺穿刺活检的患者均未经肺穿刺活检确诊为肺LCNEC，这可能与大部分周围型肺癌直接选择手术切除取大病理确诊有关，也与小标本病理检测受限有关。本组患者中有4例患者经颈部或锁骨下淋巴结活检并均确诊为肺LCNEC，确诊率达100%。可见淋巴结活检对肺LCNEC的诊断效率要明显高于活检小标本，而其创伤要明显低于开胸活检，因此有浅表部位淋巴结肿大的患者选择淋巴结活检可提高肺LCNEC诊断效率。

根据目前临床病理的发展水平，以活检小标本来区分NSCLC及SCLC难度不大，但要进一步细化评估标本的神经内分泌分化状态则富有挑战。王斌超等^[10]^认为这可能与没有足够的标本来全面了解肿瘤光镜下的神经内分泌组织形态学结构、进行免疫组化染色分析神经内分泌分化的相关抗原及计算肿瘤有丝分裂象指数（Ki67）等有关。对于肺LCNEC来说，以免疫组化评估癌组织神经内分泌状态至关重要。在本组患者中Syn、CgA和CD56的阳性表达率分别为72.7%、68.2%和68.2%，同时表达2种标志物的患者有7例，同时表达3种标志物的有8例。这与先前的报道^[11-13]^基本类似。在最新版的WHO肺癌病理分类中由于生物学行为、驱动基因、病理特征及治疗方式的相似，已将LCNEC与SCLC及TC统一分类于神经内分泌肿瘤，他们之间有相似的特征，也有特异性的区分标志^[3]^。比如TTF-1在TC中大部分呈阴性，在SCLC中阳性约85%，而在肺LCNEC中阳性约50%。本组患者中，有45.5%（10/22）患者TTF呈阳性，符合诊断标准（表 2）。病理科医生的认知水平对恶性肿瘤疾病的诊断起着至关重要的影响，这一影响尤其体现在肺LCNEC这种少见类型的疾病上。尽管总体发病率低，但国内肺LCNEC诊断率的逐渐提高肯定与病理科医生对该病的认识在不断进步有关。以我院为例，2005年-2010年共确诊2例，2010年-2013年共确诊8例，2014年-2015年10月共确诊12例。因此，针对类似LCNEC这种少见肺癌类型的认知还需要国内肿瘤科医生和病理科医生的共同进步。

自1991年Travis等^[1]^提出肺LCNEC这一肺癌新的肺癌病理类型后，有关它的治疗方案的争论一直没有停止。手术是治疗的首选，早期患者在接受手术后能明显获益，然而对是否行术后辅助化疗一直存在争论^[14-16]^。尽管目前手术是使肺LCNEC患者治愈的唯一途径，但不可否认的是即使是Ia期患者的5年生存率（54.5%）明显低于同Ia期鳞癌或者腺癌患者（89.3%）^[17]^。本研究的多因素分析提示术后接受辅助化疗的患者可有明显的生存获益。因此，我们建议即使是Ⅰ期患者，术后仍需接受辅助化学治疗。对进展期肺LCNEC目前仍推荐以铂二联为基础的化疗，推荐的方案同SCLC，但是各类报道都认为此方案对肺LCNEC的有效率和总生存不及SCLC。本组5例Ⅳ期患者有1例采用依托泊苷联合卡铂方案化疗获得部分缓解（partial response, PR），无进展生存期（progression free survival, PFS）达18.1个月，总生存22.2个月。另有4例采用NSCLC方案化疗，2例获得PR，另有2例采用紫杉类方案化疗后很快出现脑转移进展。由于例数较少，化疗方案和预后难以比较。其他的一些研究虽然样本量不大，但基本都支持应用SCLC方案化疗更有效^[18-23]^。除此以外，一些个案报道也发现个别肺LCNEC可合并表皮生长因子受体（epidermal growth factor receptor, *EGFR*）基因突变阳性，提示肺LCNEC有行基因检测的必要性，针对此类患者在一线治疗时可以应用酪氨酸激酶抑制剂（tyrosine kinase inhibitor, TKI）类药物^[24-26]^。由于发病率低，仍未见有关抗血管生成或者免疫治疗在肺LCNEC应用中的报道，但是，随着各类新药的研发，想必在不远的将来能用于治疗肺LCNEC的“武器”会越来越多。

综上所述，肺LCENE是一种具有神经内分泌分化特征的肺少见恶性肿瘤，好发于有大量吸烟史的老年男性，其临床特征缺乏特异性，确诊有赖于标本量及临床病理医师的水平。早诊断、早治疗是让患者获益的最重要手段，手术是首选的治疗方法，术后联合化疗能让患者明显获益。对晚期姑息性化疗不敏感，预后差。本研究通过分析22例LCNEC患者的临床资料及预后，可能为肺LCNEC的诊疗及研究工作提供参考。有效治疗方法的选择仍是下一步需要研究的重点，希望在今后能有更多有关肺LCNEC的科研工作开展。
